# Isolation and Characterization of Thermophilic Bacteria from Hot Springs in Republic of Korea

**DOI:** 10.3390/microorganisms10122375

**Published:** 2022-11-30

**Authors:** Yong-Jik Lee, Dariimaa Ganbat, DoKyung Oh, HyeWon Kim, Ga Eul Jeong, In-Tae Cha, Seong-Bo Kim, Gaewon Nam, You-Jung Jung, Sang-Jae Lee

**Affiliations:** 1 Department of Bio-Cosmetics, Seowon University, Chung-Ju 28674, Republic of Korea; 2 Major in Food Biotechnology, Research Center for Extremophiles and Marine Microbiology, Silla University, Busan 46958, Republic of Korea; 3 Department of Food Science and Technology, Pukyong National University, Busan 48513, Republic of Korea; 4 Library of Marine Samples, Korea Institute of Ocean Science & Technology (KIOST), Geoje 53201, Republic of Korea; 5 National Institute of Biological Resources, Incheon 22689, Republic of Korea; 6 Bio-Living Engineering Major, Global Leaders College, Yonsei University, Seoul 03722, Republic of Korea

**Keywords:** thermophiles, extracellular hydrolase, hot spring, *Geobacillus*, amylase, protease, lipase

## Abstract

Thermophiles that produce extracellular hydrolases are of great importance due to their applications in various industries. Thermophilic enzymes are of interest for industrial applications due to their compatibility with industrial processes, and the availability of the organisms is essential to develop their full potential. In this study, a culture-dependent approach was used to identify thermophilic bacteria from five hot springs in Republic of Korea. Characterization, taxonomic identification, and extracellular hydrolase (amylase, lipase, and protease) activity of 29 thermophilic bacterial isolates from the Neungam carbonate, Mungang sulfur, Deokgu, Baegam, and Dongnae hot springs were investigated. Identification based on the full-length 16S rRNA gene sequence revealed that strains belonged to the phylum *Bacillota* and were classified as *Aeribacillus*, *Bacillus*, *Caldibacillus*, *Geobacillus*, and *Thermoactinomyces* genera. It was found that 22 isolates could produce at least one extracellular enzyme. *Geobacillus*, representing 41.4% of the isolates, was the most abundant. The highest amount of proteolytic and lipolytic enzymes was secreted by strains of the genus *Geobacillus*, whereas *Caldibacillus* species produced the highest amount of amylolytic enzyme. The *Geobacillus* species producing hydrolytic extracellular enzymes appeared to be the most promising.

## 1. Introduction

Extremophiles, including thermophilic, psychrophilic, acidophilic, alkaliphilic, and halophilic microorganisms, have been studied in various fields because of their capacity to survive and function under harsh environments [1]. Among the extremophiles, thermophilic microorganisms have been generally isolated from hot springs and the deep sea of the Earth. Thermophiles are divided into moderate thermophiles (optimum growth temperature 50–60 °C), extreme thermophiles (optimum growth temperature 60–80 °C), and hyperthermophiles (optimum growth temperature 80–110 °C) [2]. They are the reservoirs for production of industrially valuable thermostable enzymes that can be used for biotechnological processes at high temperatures [3,4,5,6].

Thermostable bacterial hydrolytic enzymes have been developed and used extensively in biological processes for production, meaning that thermostability makes them highly demandable for eco-friendly industrial purposes, and extensive manufacturing and usage of thermostable enzymes has resulted from continued industrial demand [7,8,9]. Among the commercially valuable enzymes, *α*-amylases, lipases, and proteases are the major enzymes produced by thermophiles [7,10]. Their importance in biotechnological applications is significant; for example, proteases and amylases are used together in the food industry, detergent industry, and pharmaceuticals [11]. Due to their increased importance, many scientists have focused on their research to discover new thermophilic microorganisms [12,13,14].

Republic of Korea’s Hot Spring Act defines a hot spring as groundwater with temperature of 25 °C or higher with qualified standards. It is difficult to find volcanic-oriented hot springs as the Korean peninsula is known to be distant from high enthalpy geothermal energy. The area of distribution of the hot springs coincides with areas of granite, and the heat is derived from the process of radioactive decay of the granite. Hot springs with temperatures ranging from 25 °C to 78 °C are found in different regions of Republic of Korea. The low-temperature hot springs demonstrate 85.4%. The physical and chemical characteristics of Republic of Korean hot springs are the low proportion of high temperature, low mineral contents, and alkaline pH [15,16,17]. The physiochemical features of hot spring water facilitate growth of thermophiles. Consequently, research on assessment of the possibility of industrial application by isolation of thermophiles from hot spring water in Republic of Korea is necessary. This study aims to develop a continuous approach for screening, isolating, and characterizing novel thermophilic microorganisms with great biotechnological and environmental potential.

## 2. Materials and Methods

### 2.1. Sample Collection

Five hot springs in Republic of Korea were chosen to collect the sample, including the Neungam carbonate, Mungang sulfur, Deokgu, Baegam, and Dongnae Yangtangjang. The samples were collected using a pump in a sterilized sampling bottle and immediately screened for bacterial isolation. Additionally, after screening, samples were stored at 4 °C for up to 5 days.

### 2.2. Isolation of Thermophilic Bacteria

The hot spring water samples were serially diluted using a sterile 0.85% saline buffer. An aliquot of each suspension was spread on marine broth 2216 (MB; BD Difco) supplemented with 1.5% (*w*/*v*) agar (MA) plates and incubated at 60 °C for five days. Morphologically different colonies were selected, and, after several transfers using the same solid medium, pure colonies were obtained. Obtained isolates were suspended in marine broth 2216 supplemented with 10% (*v*/*v*) dimethyl sulfoxide (DMSO) and stored at −80 °C for long-time preservation.

### 2.3. Taxonomic Identification of the Isolates

The isolated strains were identified by 16S rRNA gene sequencing. The 16S rRNA identification was performed by the BIOFACT Co., Ltd. (Daejeon, Republic of Korea) using ABI PRISM 3730XL DNA analyzer (Applied Biosystems, Foster City, CA, USA). Genomic DNA of the strains was isolated using Chelex 100 Boiling Resin (BIO-RAD, Hercules, CA, USA), pro-K, and S-Taq Buffer (BIOFACT, Daejeon, Republic of Korea), followed by PCR amplification of the 16S rRNA gene by Hush Run™ cycler (BIOFACT, Daejeon, Republic of Korea) using universal primers 27F (5′-AGA GTT TGA TCC TGG CTC AG-3′) and 1492R (5′-GGT TAC CTT GTT ACG ACT T-3′). A 50 µL reaction mixture contained 5 U S-Taq DNA polymerase (BIOFACT, Daejeon, Republic of Korea), 10 mM dNTPs, and 10X S-Taq Reaction Buffer (BIOFACT, Daejeon, Republic of Korea). Identification based on the 16S rRNA sequence of the strains was analyzed using EzBioCloud Database (https://www.ezbiocloud.net/) accessed on 25 March 2022. Multiple sequence alignment was completed by the BioEdit program, and the phylogenetic tree was constructed using the Maximum Likelihood algorithm by the MEGA 6.0 program [18,19].

### 2.4. Determination of Growth Characteristics

The growth temperature range of the isolates was determined by cultivating at 45, 50, 55, 60, and 65 °C, respectively. The salt tolerance of the isolates was determined using MA medium supplemented with NaCl (*w*/*v*) concentration at 3, 6, 9, 12, and 15% incubating the plates at 60 °C. The pH tolerance was determined using MA medium with a pH range of 4.0, 7.0, and 9.0 adjusted with 1N NaOH and 1M HCl. The growth of the isolated bacteria at 60 °C on complex media was tested using nutrient agar (Difco, Sparks, MD, USA), R2A agar (Difco, Sparks, MD, USA), and tryptic soy agar (Difco, Sparks, MD, USA).

### 2.5. Determination of Hydrolytic Enzyme Activities

The hydrolytic enzyme activity of the isolated strains was tested on a marine agar medium supplemented with specific substrates. After several days of incubation at 60 °C, the enzyme activities were determined by the agar diffusion method. The amount of produced enzymes was determined as enzyme intensity (EI). EI was calculated as follows: (colony diameter + halo zone diameter)/colony diameter [20,21,22]. Each experiment was performed in triplicate.

#### 2.5.1. Amylase Activity

The amylolytic property was identified using the starch hydrolysis method. The strains were inoculated onto marine agar supplemented with 0.2% (*w*/*v*) soluble starch (Difco, USA). After incubation for 3 days at 60 °C, agar plates were treated with 1% iodine solution for 2–3 min and presence of transparent zones around the colony indicated amylase activity [23].

#### 2.5.2. Lipase Activity

Lipase activity was determined by the opaque zone around the colony as described by [24]. The strains were inoculated onto marine agar (Difco, Sparks, MD, USA) medium supplemented with 1% (*v*/*v*) Tween 80 (Sigma, St. Louis, MO, USA). Tween 80 was added separately after sterilization. After incubation at 60 °C for 3 days, lipase activity was evaluated by an opaque zone around the colony.

#### 2.5.3. Protease Activity

Protease activity was detected by inoculating the strains onto 2% (*w*/*v*) skim milk agar (Difco, Sparks, MD, USA) medium. Skim milk was added separately after sterilization. After incubation at 60 °C for 3 days, protease activity was evaluated by clear zone around the colony [25].

### 2.6. Accession Numbers

The 16S rRNA gene sequences of strains were deposited to the GenBank/EMBL/DDBJ database.

### 2.7. Deposition of Strains

All strains isolated through this study were deposited in the Microbial Value Enhancement Project, Korea Research Institute of Bioscience and Biotechnology.

## 3. Results

### 3.1. Sample Characteristics

**Figure 1:**
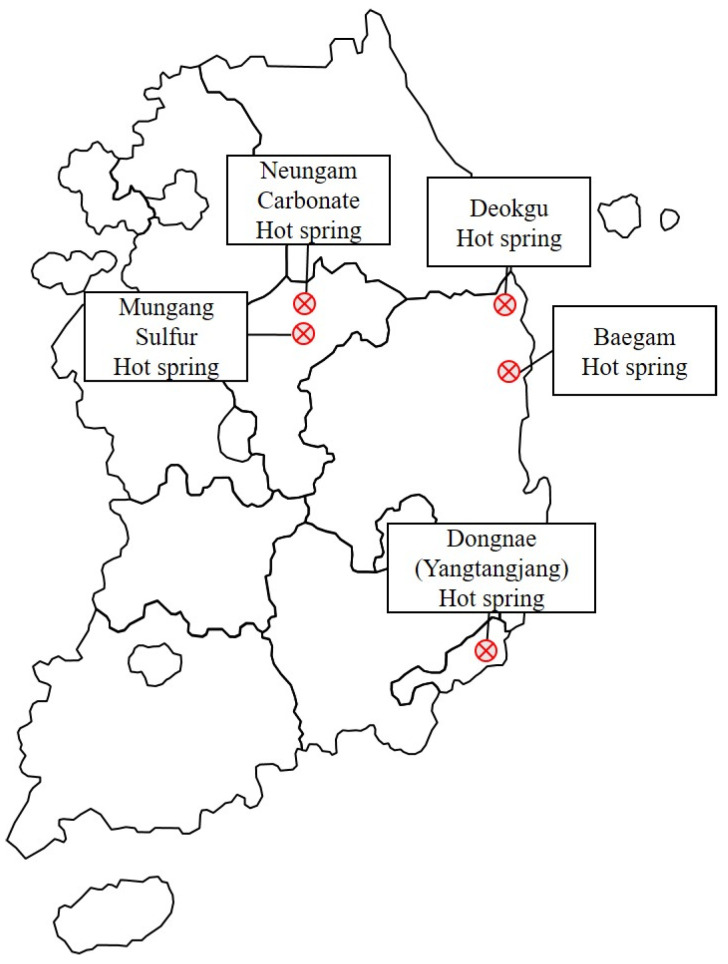
Map of the hot spring area in Korea where the samples were collected for this study. Neungam and Mungang hot springs are located in Chungju, Deokgu and Baegam hot springs are located in Uljin, and Dongnae (Yangtangjang) hot spring is located in Busan, respectively.

The samples from the five hot springs were collected in January 2017. The five hot spring sites were located in different cities in Republic of Korea (Figure 1), with a temperature range between 26 and 60 °C and pH 6.5–9.1 (Table 1).

### 3.2. Isolation and Growth Characteristics of Thermophilic Bacteria

Within the scope of this study, a total of 29 strains were isolated from hot spring water samples. The screening results showed that three strains were isolated from the Neungam Carbonate, sixteen strains from the Mungang Sulfur, two strains were isolated from the Deokgu, six strains were separated from the Baegam, and two strains from the Dongnae hot springs, respectively. All the strains showed growth on one of the complex media (nutrient agar, R2A, tryptic soy agar), which are widely used for massive cultivation of bacteria in industrial processing, meaning that newly isolated 29 thermophilic bacteria can be cultivated more easily using industrially applied commercial media. Growth of all the strains occurred between 45 °C and 65 °C, indicating that the isolated strains are thermophiles. All the strains showed growth at pH 7.0. On the other hand, any of the strains could grow at acidic (pH 4.0) and alkaline (pH 9.0) conditions. The halophilic nature of the strains showed that all the strains could tolerate 3% (*w*/*v*) NaCl concentration and 10 strains could tolerate 6% (*w*/*v*) NaCl concentration. Moreover, isolates EF60115, EF60123, and EF60155 could tolerate up to 9% (*w*/*v*) NaCl concentration. Any of the strains could tolerate up to 15% (*w*/*v*) NaCl condition (Table 2).

### 3.3. Taxonomic Identification Based on 16S rRNA Gene Sequencing and Phylogenetic Analysis

**Figure 2:**
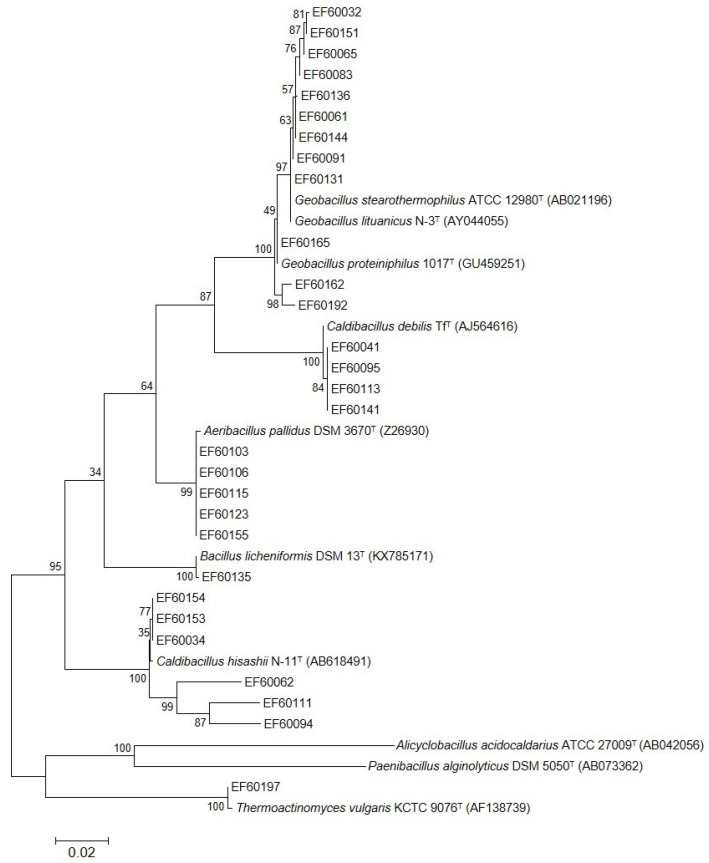
Phylogenetic analysis of 29 isolates and their closely related type strains. The phylogenetic tree was constructed based on 16S rRNA gene sequence alignments using the Maximum Likelihood method in MEGA 6.0. Bootstrap values are indicated at the branch points. The scale bar indicates a branch length equivalent to 0.02 changes per nucleotide. *Alicyclobacillus acidocaldarius* ATCC 27009^T^ and *Paenibacillus alginolyticus* DSM 5050^T^ served as outgroup.

Taxonomic identification and phylogenetic analysis of the isolates were assessed by 16S rRNA sequencing. The complete 16S rRNA gene sequences of 29 isolates were successfully amplified. A pairwise comparison result of the 16S rRNA gene sequences on the EzBioCloud server showed that there were eight different types of species (Table 3 and Table 4). As shown in Table 3, the strains belong to phylum *Bacillota*, class *Bacilli*, and order *Bacillales*. Further, twenty-eight strains belong to *Bacillaceae* (96.6%) and one strain belongs to *Thermoactinomycetaceae* (3.4%). Among the *Bacillaceae*, four genera were confirmed with five strains of *Aeribacillus* (17.2%), one strain of *Bacillus* (3.4%), eleven strains of *Caldibacillus* (37.9%), and twelve strains of *Geobacillus* (41.4%). On the other hand, one strain of *Thermoactinomyces* was identified. Phylogenetic analysis was conducted to evaluate the relationships of the isolated strains with closely related type strains (Figure 2).

### 3.4. Production of Extracellular Hydrolytic Enzymes

**Figure 3:**
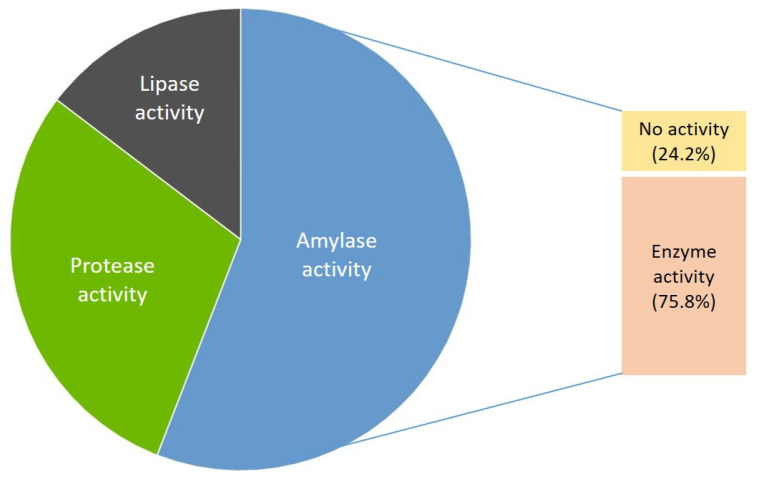
Production of extracellular hydrolase among isolates.

Bacterial isolates were screened for extracellular amylase, protease, and lipase activity. Among the isolates, at least one extracellular enzyme activity was detected in 22 strains (75.9%). Amylase activity was detected in nineteen strains (65.5%), protease activity in ten strains (34.5%), and lipase activity in five strains (17.24%) (Figure 3). The degree of hydrolytic capacity for degrading a certain substrate was evaluated and shown as enzyme intensity (EI) to show which of the isolates produced larger amounts of the enzymes (Table 5). The amylolytic activity was the most common among isolates with the highest amylase activity in strains EF60154 (EI 4.02), EF60094 (EI 3.33), EF60111 (EI 3.16), and EF60034 (EI 3.1) and strains EF60165 (EI 3.67), EF60131 (EI 3.12), EF60136 (EI 3.09), and EF60061 (EI 3.08). The highest protease and lipase activity was detected in strains EF60192 (EI 10.71) and EF60131 (EI 3.46), respectively. Two strains, EF60083 and EF60192, produced all the tested hydrolytic enzymes.

## 4. Discussion

The region of hot springs that are habitats for thermophiles is limited to a restricted number of sites. In Korea, there are about 400 hot springs, many recognized as having therapeutic and medicinal effects. In these environmental conditions, living organisms have to cope with harsh temperatures and the low availability of nutritional compounds. Biodiversity in these environments is low, but some microorganisms can adapt to these conditions by developing survival strategies.

Bacterial strains isolated in this study are thermophiles that showed growth at relatively high temperatures. One of the important potentials of thermophiles is their productive capacity of thermostable enzymes. Applications of enzymes in industries are numerous; for example, hydrolases (amylases, lipases, and proteases, etc.) account for over 75% of all enzymes produced on a commercial scale. These thermostable hydrolytic enzymes can be extracted from thermophiles because they have many desirable properties, including the capacity to reduce the probability of microbial contamination in large-scale fermentation and the capacity to function for extended periods of time [26,27]. This reason leads to intensive and extended studies to perform fully for investigating such promising microorganisms. The majority (75.8%) of the thermophilic strains produced at least one extracellular hydrolase (Table 4). The amylolytic activity was the most common in isolates with 19 (65.5%) strains. The highest amylase activity was produced by isolate EF60154 from the Baegam hot spring, which was identified as *Caldibacillus hisashii*. Proteolytic activity was detected in 10 (34.5%) strains, with the highest protease activity in isolates EF60192 and EF60165, which were identified as *Geobacillus proteiniphilus*. Lipolytic activity was less common among isolates with 5 (17.2%) strains, producing very small amounts. The highest lipase activity was detected in the EF60131 strain, which was identified as *Geobacillus lituanicus.* Isolates EF60192 and EF60083 that were identified as *Geobacillus proteiniphilus* and *Geobacillus stearothermophilus*, respectively, produced all the tested enzymes. Among the isolates, eight strains could produce two extracellular hydrolases. On the other hand, seven strains did not show any enzyme activity. There are numerous biotechnological uses for hydrolases; each one necessitates certain enzyme properties in terms of specificity and thermostability [28]. There may be differences in enzyme production depending on the environmental factors and chemical characteristics of the water samples [29]. Most of these promising thermophilic bacterial isolates belong to the genus *Geobacillus*. Adaptation of *Geobacillus* spores to environmental stress and meeting their basic nutritional requirements for growth may be the reason for the occurrence of these species in all five hot springs [30].

The results of this study will achieve great significance in terms of securing the diversity of microbiological resources in Korea, and it is expected that a new strain can be found for production of hydrolytic enzymes in water samples around domestic hot spring regions and basic biological materials for research on bioengineering in food and cosmetics industries.

## Figures and Tables

**Table 1:** Hot spring properties.

Hot Spring	Temperature (°C)	pH	Chemical Composition	Location (GPS)
Neungam Carbonate	26.2–27.3	6.5–6.61	Carbonate (Na, K, Ca, Mg, Li, Sr, HCO_3_, CO_2_, Cl, SO_4_^2−^, F, dissolved SiO_2_, Fe, Mn, Zn, Cu, Pb, Al, S)	37°05′36.5″ N 127°48′06.6″ E
Mungang Sulfur	26.4–28.3	8.94–9.11	Na, Ca, K, SO_4_^2−^, Cl, H₂S, HCO_3_, SiO_4_, Zn, CO_3_, F, free H₂CO_3_	36°53′08.0″ N 127°57′13.9″ E
Deokgu	42.3	8.49	K, Na, Ca, Mg, Cl, SO_4_, CO_3_, SiO_2_, F, Li, Sr, Zn, Al, Na-HCO_3_	37°04′43.6″ N 129°17′03.4″ E
Baegam	45.9	8.93	K, Na, Ca, Mg, SiO_2_, Li, Sr, Fe, Na-CO_3_	36°43′15.0″ N 129°20′22.2″ E
Dongnae	59.4	7.6	Na, K, Ca, Mg, Li, Sr, O_3_, Free CO_2_, CI, SO_4_, F, SiO_2_, Fe, Mn, Zn, Cu, Pb, Al	35°13′15.9″ N 129°04′51.5″ E

**Table 2:** Growth characteristics of aerobically cultured thermophilic bacteria isolated from the diverse hot springs in Republic of Korea.

No	Source	Isolate Number				Growth Characteristics												
			Media			NaCl %					pH			Temperature				
			NA	R2A	TSA	3	6	9	12	15	4.0	7.0	9.0	45 °C	50 °C	55 °C	60 °C	65 °C
1	Neungam Carbonate Hot spring	EF60032	+	+	+	+	–	–	–	–	–	+	–	+	+	+	+	+
2		EF60034	+	+	+	+	+	–	–	–	–	+	–	+	+	+	+	+
3		EF60041	–	+	+	+	–	–	–	–	–	+	–	+	+	+	+	+
4	Mungang Sulfur Hot spring	EF60061	+	+	+	+	–	–	–	–	–	+	–	+	+	+	+	+
5		EF60062	–	+	+	+	–	–	–	–	–	+	–	+	+	+	+	+
6		EF60065	+	+	+	+	–	–	–	–	–	+	–	+	+	+	+	+
7		EF60083	+	+	+	+	–	–	–	–	–	+	–	+	+	+	+	+
8		EF60091	+	+	–	+	–	–	–	–	–	+	–	+	+	+	+	+
9		EF60094	+	+	+	+	+	–	–	–	–	+	–	+	+	+	+	+
10		EF60095	–	+	–	+	–	–	–	–	–	+	–	–	+	+	+	+
11		EF60103	+	+	+	+	+	–	–	–	–	+	–	+	+	+	+	+
12		EF60106	+	+	+	+	+	–	–	–	–	+	–	+	+	+	+	+
13		EF60111	+	+	+	+	+	–	–	–	–	+	–	+	+	+	+	+
14		EF60113	–	+	+	+	–	–	–	–	–	+	–	+	+	+	+	+
15		EF60115	+	+	+	+	+	+	–	–	–	+	–	+	+	+	+	+
16		EF60123	+	+	+	+	+	+	–	–	–	+	–	+	+	+	+	+
17		EF60131	+	+	+	+	–	–	–	–	–	+	–	+	+	+	+	+
18		EF60135	+	+	+	+	–	–	–	–	–	+	–	+	+	+	+	+
19		EF60136	+	+	+	+	–	–	–	–	–	+	–	+	+	+	+	+
20	Deokgu Hot spring	EF60141	+	+	+	+	–	–	–	–	–	+	–	+	+	+	+	+
21		EF60144	+	+	–	+	–	–	–	–	–	+	–	+	+	+	+	+
22	Baegam Hot spring	EF60151	+	+	+	+	–	–	–	–	–	+	–	+	+	+	+	+
23		EF60153	+	+	+	+	+	–	–	–	–	+	–	+	+	+	+	+
24		EF60154	+	+	+	+	+	–	–	–	–	+	–	+	+	+	+	+
25		EF60155	+	+	+	+	+	+	–	–	–	+	–	+	+	+	+	+
26		EF60162	–	–	–	+	–	–	–	–	–	+	–	+	+	+	+	+
27		EF60165	–	+	–	+	–	–	–	–	–	+	–	–	–	–	+	+
28	Dongnae Hot spring	EF60192	–	+	+	+	–	–	–	–	–	+	–	+	+	+	+	+
29		EF60197	–	+	–	+	–	–	–	–	–	+	–	+	+	+	+	+

NA: nutrient agar, MA: marine agar Difco 2216, TSA: tryptic soy agar, +: well–growth, –: no growth.

**Table 3:** Phylum analysis of aerobically cultured thermophilic bacteria isolated from the diverse hot springs in Republic of Korea.

Phylum	Class	Order	Family	Genus	Closest Species	Strains
*Bacillota*	*Bacilli*	*Bacillales*	*Bacillaceae*	*Aeribacillus*	*Aeribacillus pallidus*	5
				*Bacillus*	*Bacillus licheniformis*	1
				*Caldibacillus*	*Caldibacillus debilis*	4
					*Caldibacillus hisashii*	6
				*Geobacillus*	*Geobacillus lituanicus*	4
					*Geobacillus proteiniphilus*	3
					*Geobacillus stearothermophilus*	5
			*Thermoactinomycetaceae*	*Thermoactinomyces*	*Thermoactinomyces vulgaris*	1

**Table 4:** Taxonomic identification of the isolates based on 16S rRNA gene sequences.

No	Isolate Number	Closest Strain	Closest Strain Number	Similarity (%)	Deposited Number	GenBank Accession Number
1	EF60032	*Geobacillus stearothermophilus*	NBRC 12550	98.81	NMC4-B346	ON365874
2	EF60034	*Caldibacillus hisashii*	N-11	99.79	NMC4-B347	ON365877
3	EF60041	*Caldibacillus debilis*	Tf	99.79	NMC4-B348	ON365879
4	EF60061	*Geobacillus lituanicus*	N-3	99.38	NMC4-B352	ON365885
5	EF60062	*Caldibacillus hisashii*	N-11	99.93	NMC4-B353	ON365925
6	EF60065	*Geobacillus stearothermophilus*	NBRC 12550	99.3	NMC4-B355	ON365932
7	EF60083	*Geobacillus stearothermophilus*	NBRC 12550	99.49	NMC4-B356	ON365961
8	EF60091	*Geobacillus lituanicus*	N-3	99.72	NMC4-B357	ON366319
9	EF60094	*Caldibacillus hisashii*	N-11	99.86	NMC4-B359	ON366407
10	EF60095	*Caldibacillus debilis*	Tf	99.79	NMC4-B360	ON366406
11	EF60103	*Aeribacillus pallidus*	KCTC 3564	100	NMC4-B361	ON366400
12	EF60106	*Aeribacillus pallidus*	KCTC 3564	99.86	NMC4-B362	ON430523
13	EF60111	*Caldibacillus hisashii*	N-11	99.86	NMC4-B363	ON430527
14	EF60113	*Caldibacillus debilis*	Tf	99.79	NMC4-B364	ON430529
15	EF60115	*Aeribacillus pallidus*	KCTC 3564	98.71	NMC4-B365	MW132409
16	EF60123	*Aeribacillus pallidus*	KCTC 3564	99.86	NMC4-B366	ON430531
17	EF60131	*Geobacillus lituanicus*	N-3	99.65	NMC4-B367	MW131318
18	EF60135	*Bacillus licheniformis*	ATCC 14580	99.73	NMC4-B370	ON430534
19	EF60136	*Geobacillus lituanicus*	N-3	99.39	NMC4-B371	ON430536
20	EF60141	*Caldibacillus debilis*	Tf	99.66	NMC4-B372	MW131319
21	EF60144	*Geobacillus stearothermophilus*	NBRC 12550	99.39	NMC4-B373	MW131115
22	EF60151	*Geobacillus stearothermophilus*	NBRC 12550	99.02	NMC4-B374	ON430581
23	EF60153	*Caldibacillus hisashii*	N-11	99.79	NMC4-B375	ON430584
24	EF60154	*Caldibacillus hisashii*	N-11	99.79	NMC4-B376	ON430575
25	EF60155	*Aeribacillus pallidus*	KCTC 3564	99.86	NMC4-B377	ON430576
26	EF60162	*Geobacillus proteiniphilus*	1017	98.8	NMC4-B378	MW131211
27	EF60165	*Geobacillus proteiniphilus*	1017	99.73	NMC4-B380	ON430578
28	EF60192	*Geobacillus proteiniphilus*	1017	98.71	NMC4-B381	ON430582
29	EF60197	*Thermoactinomyces vulgaris*	KCTC 9076	99.66	NMC4-B382	MW131315

Culture collection numbers: *Caldibacillus hisashii* N-11^T^ (NBRC 110226); *Caldibacillus debilis* Tf^T^ (DSM 16016); *Geobacillus lituanicus* N-3^T^ (DSM 15325); *Geobacillus proteiniphilus* 1017^T^ (KCTC 33986).

**Table 5:** Amount of extracellular hydrolases produced by thermophilic isolates expressed as EI.

No	Isolate Number	Enzyme Activity			Enzyme Intensity (EI)		
		Amylase	Lipase	Protease	Amylase	Lipase	Protease
1	EF60032	+	–	–	2.33 ± 0.06		
2	EF60034	+	–	–	3.1 ± 0.15		
3	EF60041	–	–	–			
4	EF60061	+	–	+	3.08 ± 0.1		4.65 ± 0.55
5	EF60062	+	–	–	2.92 ± 0.17		
6	EF60065	+	+	–	2.32 ± 0.09	2.22 ± 0.02	
7	EF60083	+	+	+	2.26 ± 0.07	2.12 ± 0.02	2.18 ± 0.02
8	EF60091	+	–	–	2.92 ± 0.22		
9	EF60094	+	–	–	3.33 ± 0.17		
10	EF60095	–	–	–			
11	EF60103	–	–	–			
12	EF60106	–	–	–			
13	EF60111	+	–	–	3.16 ± 0.18		
14	EF60113	–	–	+			2.69 ± 0.38
15	EF60115	–	–	–			
16	EF60123	–	–	–			
17	EF60131	+	+	–	3.12 ± 0.32	3.46 ± 0.23	
18	EF60135	+	+	–	2.15 ± 0.06	2.25 ± 0.1	
19	EF60136	+	–	+	3.09 ± 0.07		4.46 ± 0.66
20	EF60141	–	–	+			2.18 ± 0.06
21	EF60144	+	–	+	2.16 ± 0.06		3.65 ± 0.47
22	EF60151	+	–	–	2.2 ± 0.08		
23	EF60153	+	–	–	3.1 ± 0.08		
24	EF60154	+	–	–	4.02 ± 0.34		
25	EF60155	–	–	–			
26	EF60162	+	–	+	2.59 ± 0.34		3.3 ± 0.48
27	EF60165	+	–	+	3.67 ± 0.45		7.88 ± 1.07
28	EF60192	+	+	+	2.54 ± 0.15	2.92 ± 0.17	10.71 ± 2.65
29	EF60197	–	–	+			2.51 ± 0.05

+: enzyme activity, –: no activity.

## Data Availability

Not applicable.
